# From Automation to Symmation: Ethnographic Perspectives on What Happens in Front of the Screen

**DOI:** 10.1007/s42438-022-00350-z

**Published:** 2022-10-29

**Authors:** Nadine Wagener-Böck, Felicitas Macgilchrist, Kerstin Rabenstein, Annekatrin Bock

**Affiliations:** 1 Institute for European Ethnology/Folklore Studies, Kiel University, Kiel, Germany; 2 Leibniz Institute for Educational Media | Georg Eckert Institute, Freisestr. 1, 38118 Brunswick, Germany; 3 Institute for Educational Science, University of Göttingen, Goettingen, Germany

**Keywords:** Automation, Classroom practices, Educational technology, Ethnography, Human–computer interaction, Platforms

## Abstract

The work of automation in education is not automatic but needs to be ‘done’. Grounded in an ethnographic study which followed a Grade 9/10 class through their daily activities in a ‘regular’ high school for a year, this paper asks how automation is enacted by students and teachers, and what these practices imply for forms of knowledge and relationality. Inspired by feminist technoscience, and drawing on recent work on everyday automation, the paper suggests that the ‘auto-’ of automation in practice is very often more of a ‘sym-’, a ‘with’, in which students and machines co-produce something that looks like automation. Rather than ‘automation’, observing practices in classrooms shows practices of ‘symmation’. The paper elaborates on symmation scenes of realigning, revising and reworking relations. Automation is, in these scenes, deeply embedded in social relations, involving the processing of ability, difference and hierarchy. Rather than the industry hype of automation, these sets of socio-technical practices alert us to the messy, non-linear, contested, warm realities of education (and not just learning) in schools today. The paper identifies specific aspects of how these socio-technical realities impact knowledge and teacher-student relations.

## Introduction

Sympoiesis﻿ is a simple word; it means ‘making-with’. Nothing makes itself; nothing is really autopoietic or self-organizing. In the words of the Inupiat computer ‘world game’, earthlings are never alone. (Haraway 2016)^1^

A key promise of automation is that machines will take tasks off our (human) hands, and complete them by themselves. In a very basic sense, the ‘auto-’ of automation refers to operating by yourself, independently, without the help of people. In this paper, drawing on observations of apparently automatic technology-driven moments in classrooms, we suggest that the ‘auto-’ of automation in educational practice is very often more of a ‘sym-’, a ‘with’ (Haraway 2016: 58), in which students, teachers and machines co-produce something that looks like automation. ‘Automation’, as enacted in plural situated sociomaterial assemblages, is perhaps better referred to as ‘symmation’.

Critical approaches to automation and education have analysed the impact and implications of, for instance, facial recognition systems (Selwyn 2022b), automated essay feedback (Dixon-Román et al. 2020), school monitoring (Hartong and Förschler 2019), predictive analytics (Jarke and Macgilchrist 2021) or bioinformatic technologies (Williamson 2020). The promise of automation orients to efficient ‘teacher-light’ tuition (Bayne 2015) and ‘friction-free’ interaction (Gilliard 2018). A key concern across these analyses is that the mechanisms of automation introduced through platforms, apps, software and hardware constrain the capacity of teachers, learners, school leaders or others to act beyond the limited roles configured by these automated technologies (Perrotta et al. 2020). Automation is thus seen as shifting pedagogical priorities, e.g. with regard to teacher’s tasks (Popenici and Kerr 2017; Gallagher and Breines 2021), with the potential to ‘automat[e] educational judgement’ (Selwyn et al. 2021), to orient students more to ‘interpassivity’ than interactivity (Macgilchrist 2012), and to render ‘the labour of pedagogy more machine-like’ (Perrotta et al. 2020: 3), when reconfiguring teachers’ work with, for instance, tasks requiring teachers to ‘synchronise effectively with the platform’ (12).

Yet at the same time, a research programme into the ‘scrappy realities’ (Selwyn and Jandrić 2020) of automation in daily life, which are anything but friction-free, is emerging, which seeks to redefine the analytical focus. Writing about ‘everyday automation’, for instance, Pink et al. (2022) suggest that critical data and algorithm studies tend to adopt a similar universalising and techno-centric approach as the systems or narratives that they analyse. These studies ‘become complicit in making and sustaining the very paradigms and logics that they critique if they do not acknowledge the situatedness of processes of power’ (Pink et al. 2022: 8). This situatedness, they argue, can be explored by ‘rehumanising automation’, i.e. engaging closely with the ‘ethnographic realities’, the ‘actual experiential and social and political encounters, meanings and implications’ of automated technologies in everyday life (Pink et al. 2022: 5, 8, 17). Similarly, Smith and Fressoli’s (2021) ‘post-automation’ critiques the essentialism of writing about automation—from both industry advocates and post-capitalist proposals for ‘fully automated luxury communism’ (Bastani 2019). The concept of post-automation aims to capture the human capabilities of those subverting automation by radically repurposing automation technologies, for instance, through commons-based peer-production, platform cooperatives or the right to repair (Smith and Fressoli 2021). More broadly, much of the writing about ‘postdigital’ practices emphasises the heterogeneity, muddiness and noise around designing and using automated technologies (e.g. Jandrić et al. 2018; Macgilchrist 2021).

These approaches centre the creativity, care and repair that goes into making ‘automation’ seem to work autonomously and seamlessly in everyday, mundane settings. The use of an adaptive learning system for maths in a Danish primary school, for instance, highlights the unruly mess of using apparently automated feedback systems in classrooms, with some students stuck, others rushing ahead, yet others disappearing to the toilets and the teacher constantly moving across the room to assist students with the system (not the maths), trouble-shooting, logging in, dealing with conflicts, etc. (Cone 2021). Reflecting in interviews on a similar adaptive maths system in Germany, teachers describe how they adapt the system, throwing out the majority of the automated aspects in order to embed the system in their pedagogical priorities, and their relations of care for their students, e.g. to ensure that students understand that making mistakes is ok, and an important part of learning (Macgilchrist et al. 2022). When teachers and union representatives discuss the implementation of automated facial recognition software for recording student attendance in Australian classrooms, they reject the assumption that the attendance roll call in class is not deeply pedagogical work, describing how teachers use the roll call as a commencement activity, a ceremonial process, a welcoming that can lead to conversations, a first indication of how the class is feeling that day, and ‘a whole bunch of the relational processes’ that ‘can’t be discounted’ (interviewee in Selwyn 2022b: 81).

Contributing to this emerging interest in the mundane, everyday settings of automation, this paper centres a secondary-school classroom. Our focus is primarily on students, and their use of the most everyday of automated technologies, such as search engines, translation websites and quizzes. Here, rather than asking ‘what automation does to people’, we initially aimed to explore ‘what people do with automation’ (Pink et al. 2022: 4) in school settings. Yet even this question implies that ‘automation’ is a thing ‘out there’ which our analysis was in danger of reifying. As our fieldwork progressed, and we observed the ‘making’ of automation (see below), we thus refocused our analysis. This leads us to argue that, when automation is enacted in the classroom, it unfolds more as *sym-mation*, in the sense of ‘made-with’, than simply *auto-mation*. The following sections first outline the study and methodology from which our analysis emerges before exploring three enactments of ‘symmation’ in the classroom. The final section reflects on key issues emerging from the analysis, including the destabilising of clear-cut roles and predetermined practices in front of the screen, and the specifics of *these* students in *this* classroom. It also opens further questions for future research on processes that look like automation in education.

## Ethnographies of Sociomaterial Classroom Practices

This paper draws on vignettes stemming from an ethnography in which we spent one year with a focal class at an integrated comprehensive school in Germany. In contrast to ethnographies that focused on schools identified as particularly high-tech, innovative, or ‘disruptive’ (Sims 2017), we selected a ‘regular’ school in this respect with one class as the case study for our research. We joined the 28 students and their pair of class teachers in their ninth grade and continued with them to tenth grade. Each student has the same type of Android-based tablet, which they loan from the school.

The ethnographic fieldwork in the school began early in 2021, and thus, in a phase of the Covid-19 pandemic in which video conferences during emergency remote teaching shaped everyday school life. From January to April 2021 the class met in a morning video conference, which one of the research team attended. The school soon shifted back to in-person attendance but split each class into two half-classes who attended on alternate days. In the early summer of 2021, the entire class came together again. From May 2021 to January 2022 two researchers conducted participant observation in lessons and hung out with students during breaks. All in all, our written field notes, which are supplemented by photos, screenshots, and collections of learning materials, describe 55 days in class. We also shadowed the two class teachers’ working days and participated in staff meetings in which they discussed working with digital technologies. Alongside numerous informal conversations, we conducted semi-structured interviews: seven interviews with students from the class observed, two interviews with each class teacher, six interviews with further subject teachers, including music, physics and English, two interviews with the school’s technical assistant and one with the school’s director. From the beginning, we were also invited into the school’s learning management system and accessed apps and systems used in class, which resulted in further field notes including screenshots. Informed consent was obtained from students and their guardians, from teachers and school leaders. Ethical approval was granted by the regional school board, the body which approves research in schools in this federal state in Germany.

As a whole, the project explores how digital technologies are woven into ‘learning cultures’ (Rabenstein et al. 2022a). Oriented towards practice theory (Schatzki 1996) the analysis of learning cultures focuses on social practice in classrooms as multi-modal instances, taking language, materiality, corporeality, temporality and spatiality into account. The approach considers human activity to be interwoven with material arrangements, and thus adopts a sensitivity for artefacts and things (Rabenstein 2018; Rabenstein et al. 2022b). In line with traditional modes of ethnography, we maintained an exploratory view regarding technologies and practices (Breidenstein et al. 2020). But in addition, we adopted perspectives from social-material ethnography combining long-term participant observations, ethnographic interviews and the analysis of artefacts (tablets, handouts, etc.) to trace lessons and school life as sociomaterial assemblage (Cameron et al. 2020; Röhl 2015), which means we consider ‘material effects and social relations concurrently, and in a way which allows us to see them as mutually emergent rather than hierarchical and distinct’ (Bayne 2015). This approach proved especially fruitful as the ethnographic material condensed and we identified ‘rich points’ (Agar 2006) to pursue and deepen. Rich points, as those moments which confuse, surprise, and seem to need new explanations, led us to an iterative research process in which the research team discussed individual scenes to find core concerns related to automation, searched for further scenes in the fieldnotes, and distilled key issues which were relevant across scenes. A small selection of scenes was then identified for lengthier discussion in this paper.

During fieldwork in the school, we spent a lot of time looking over students’ shoulders at their digital devices (tablets). What at first glance hardly seemed like automation—given contemporary discussions of AI or automated decision-making systems (ADMs)—caught our interest by its very inconspicuousness. In the sense of ‘everyday automation’, the use of Google Translator, Google Search or quizzes and other apps proved to be regular practices in the everyday life of the class during lessons and breaks. As noted above, these small and incidental practices in front of the screen are rarely part of academic analysis and reflection, yet they can open up crucial insights into the creativity, care and repair involved in enacting automation together, i.e. symmation, in today’s schools. These practices go some way towards contesting dominant narratives about the potentials or dangers of automated futures.

## Everyday Automation in Education

The following analyses of these apparently mundane, yet deeply generative moments bring forth small but relevant aspects of how school, teaching and learning are changing alongside new technologies. Our goal is not to provide a typology, heuristic, conceptual map or universalising theory of symmation. Instead, rooted in ethnographic traditions, we aim to tell stories about the complexities of situated practice. These stories will, we hope, resonate with other research on lived experiences with automation, and thus, re-orient priorities and expectations of these kinds of technologies. Since we pick up the well-established observation here that social life is messy, it becomes interesting to explore how mess plays out in specific situations as auto-/symmation and how these moments work. In order to avoid the temptation to tidy the heterogeneous observations into a heuristic or typology, we present these stories as a ‘list’, which is by definition provisional and unfinished, ‘since there’s no reason to suppose that any list will ever be complete’ (Law et al. 2013: 3). The following three vignettes address realigning, revising and re-working relations.

### Realigning

A key format for mundane, ‘low-level’ automations in everyday school life involves Google. Search, for instance, was an unspectacular and unpretentious part of how students dealt with their tasks. Students did not always simply accept the top search hits or the automated query completions, as the following fieldnote from a religious education class illustrates.

‘Mara’^2^ types into Google’s search box. The automatic phrase completions that are displayed to her as she types do not seem to correspond to what she is looking for, because she deletes what she has typed several times, and starts again from the beginning. (Fieldnote_class 30)

The task set for the students in this lesson was to find out about the difference between ethics and morality. The vignette describes Mara’s search using Google Search on her school-issued tablet. Attending to what happens at the ‘front-end’ of autocomplete predictions, we see Mara’s multiple rewriting attempts. As she is typing, reading the prediction, deleting the result, typing again, reading the prediction and so on, it seems that the automation of information search is part of a broader bundle of practices which hints at diverse phenomena not yet fully in focus when thinking in concepts such as ‘low-level’ or ‘high-level automation’ (Manovich 2013: 138). Google describes ‘autocomplete’ as ‘a feature within Google Search designed to make it faster to complete searches that you’re beginning to type’ and as Google’s ‘best predictions of the query you were likely to continue entering’, based on ‘real searches that happen on Google’, ‘common and trending’ searches ‘related to your location and previous searches’ (Sullivan 2018). However, Mara’s repeated deletion and restarting mark her process of realigning away from these ‘best predictions’ towards other queries, other search terms, other concerns and priorities.

What is striking is the temporality unfolding in the situation. In contrast to the narrative of automation as time- and therefore labour-saving, Mara’s search is a meandering act. The back and forth of the cursor expresses the act of sorting out predictions not fitting into Mara’s idea of what might lead to the information she can work with to complete the task set by the teacher. This challenges the idea of automation as anticipatory or predictive in general, and automated information research in particular, because the auto-completion fails to anticipate the classificatory framework that Mara brings to the search. Google’s ability to ‘make it faster to complete search’ bangs its nose against Mara’s ability to reflect and realign her search towards other ends and to thereby slow search down. Given that she is searching for information to work on a school task it might not seem surprising that autocomplete does not work smoothly, since its predictions are partially based on the analysis of data generated through previous queries. However, for Mara automation here in no way proves to be a decrease in work, and thus, a release of time and potential. If we slow down the scene, it almost looks like a wrangling over the power to define the search: By struggling ‘against’ the automatically generated predictions, Mara reasserts her role in completing the search. She does not entirely ignore the predictions. But nor does she hand over the search tasks to the autocomplete. Instead, she realigns the search, operating (sympoietically) ‘with’ Google’s autocomplete algorithms to trial several options, until—together—they identify a search term sufficiently useful to find the information Mara considers relevant.

### Revising

The friction becomes even clearer in the following interview excerpt, when a teacher, we’ll call her ‘Carla Oras’, describes her experiences when a student, ‘Noah’, created a Kahoot, an online quiz for game-based learning, for his science presentation. He prepared his presentation and the Kahoot in the local language, German.

Carla: If a student says they want to create one [a Kahoot!] (.) I say send it to me beforehand and then (.) I had that for a presentation, I remember Noah, I corrected it like (.) five times and because it was in English, so every time you saved it, it became wrong somehow ... there were just wrong words. ... It was madness. ... I told him the things that were wrong and every time something else came up that was wrong. ... Then someone told me that it was because it was originally in English and that’s why it was always erm incorrectly corrected. It was a presentation in science [and there were] all these nonsense words or words were missing or, so it was so completely (.) well so I identified that (sighs) and then when he showed it here it was still wrong, right? I don’t know how often I wrote back and forth with him about what was wrong. Well, he is super, he always wants to do everything correctly and he was very enthusiastic, right? But until I understood I was wondering why on earth has he got a mistake there again? And that it wasn’t him, it wasn’t him, but that it was because of the program and the app. (Interview_teacher_08)

While talking about integrating apps and devices into her daily teaching routines, Carla tells us this story of how Kahoot developed a life of its own. One of her students created a quiz for his science presentation. The spelling in the quiz is all over the place, there are nonsense words and misspellings. Carla tells us that she corrected it with him in multiple iterations, but his final presentation in class still contained some spelling mistakes. Only when another student alerted her to the probable cause—that the autocorrect is set to the programming language, English, and was thus correcting German words to English words that are similarly spelt—did she understand why this student, who she describes as someone who always wants to get everything right, was making these kinds of repeated mistakes.

Three interlinked forms of recursive revision become visible in this scene: Kahoot offers its automatic revision of what it detects as misspelt words. The teacher’s revisions consist of alerting the student to the spelling mistakes that she has noticed. And the student makes manual revisions by correcting those mistakes, simultaneously generating—sympoietically with Kahoot—further mistakes. This iteration happens several times, with revision leading to mistakes leading to revision leading to mistakes. Carla’s reflection includes an assessment of Noah’s learning capacities. But unlike the reconfigurations of teacher-student relations that have been identified in research on dashboards, learning systems or other automatic feedback systems which aim to provide teachers with insights on their students’ learning (see above), Carla integrates Noah’s mistakes into her prevailing view of his ‘enthusiasm’ and his desire to ‘get things right’. Her assessment of his ability does not seem to be affected by the auto-/symmatic mistake generation, but instead, she wonders why he continues to present a flawed presentation.

Presentations and other tasks are not usually corrected by the teacher in advance but are done for assessment in class. In this narrative, Carla invests extra time and effort into helping to revise Noah’s work. Key for us, given scholarly concerns about automation, is that despite the potential for Kahoot’s autocorrect to sabotage not just the student’s attempt to achieve a good result, but also the teacher’s view of her student, Carla’s narrative integrates the autocorrections into existing relations of respect. She describes the process as ‘madness’. But this is not the madness in which automated systems usurp her impression of Noah or in which her pedagogy becomes more machine-like. Nor is this an instance of ‘auto’-mation making work more effortless, efficient or seamless. Instead, it is the sociomaterial ‘with’ of ‘sym’-mation across the student–teacher-algorithmic-linguistic work of revising that creates the presentation in its final form and the story of autocorrect-madness which Carla relates to us.

### Re-working

During our year-long stay in class, we attended several ‘project days’ in which students worked on cross-subject issues. The school has regular project days to dedicate time to specific learning settings to foster self-determined learning. In addition, due to the Covid-19 pandemic, the Ministry of Education had opened up possibilities for schools to go beyond conventional subject-specific instruction to help students readjust after the schools had been closed for months. The following vignette, the final of three, presents a longer scene from one of these project days. The students were supposed to be testing and improving their basic skills in German, English and History.

One of the class’s two tutors and their history teacher, ‘Markus Friedemann’, had prepared some materials and apps for the day. One learning website attracted the most attention: ‘schlaukopf.de’ (smartypants.de, our translation). We could see several students clicking through its topics. The website provides exercises for different year groups, ability levels and subjects. Each topic consists of a series of multiple-choice questions. Questions appear one by one, with users selecting one of the answers. If the answer is right, a green tick appears and a chime sounds, if it is wrong, a red cross appears and a different tone chimes. In both cases, the correct answer turns green. To the right of the multiple-choice answers, a segment shows an automated grade for the user’s responses. Starting with a grade based on the first answer, each following response has an impact on the grade, which appears in red below a bar measuring your progress (with the correct answers marked by green and wrong answers marked by red). With one wrong answer, the grade plummets. Subsequent correct answers increase the grade incrementally. This kind of performance measurement became crucial when one student came to the front of the classroom.

Ben sits down at the table directly opposite the teacher’s table where the teacher is sitting. Ben has taken his tablet with him. ‘Mr Friedemann’, he starts in his charming and teasing way, which is always a little cheeky. ‘Ben’, Mr Friedemann looks up at him as he sets aside the mobile phone in his hand. ‘Mr Friedemann’, Ben repeats, trying to give his voice a certain weight and thus, somehow playfully giving the situation seriousness and significance. He wants to test the tutor’s basic skills, he announces with a straight back and the tablet in his hand. Meanwhile his neighbour at the table, Johannes, comes up from behind. Anton, another student, sits down next to Ben. Ben explains that it is about history, he would expect a 1.0 [an A], because Mr Friedemann teaches them the subject. Mr Friedemann is moderately enthusiastic. He rests both arms on the teacher's desk, yes, he studied history, he sighs. A moment later he is confronted with the first question. (Fieldnote_class_35)

Ben goes to the front, addresses his teacher, and assigns him the role intended by the learning website for learners, i.e. in this school context primarily the students’ role. The teacher does not refuse but seems weary as he takes up the challenge. What happens here is a reworking of the relations in class through playing with an automated grading system. By initiating the challenge and changing his voice, Ben takes on the role of a teacher or moderator. The teacher, as the one who will respond to questions and be graded by the system in real-time for these responses, takes on the role of student or test-taker. The question-and-answer reversal could have the quality of a quiz-master and television show celebration, but the grading adds an extra dimension, especially on this project day, during which students are preparing for tests which will have significant consequences for their futures. The scene continues:

‘Mr Friedemann, man, you got yourself down to a 2.3!’ Ben exclaims, his voice sounding like a quiz-master, in keeping with a certain game nature, which is, as I note, quite remarkable, because the website design is very restrained in this respect, compared to other apps. Ben reads out the questions, and Mr Friedemann responds. Back and forth it goes. One time, the history teacher points out that the sources for these questions are not always unambiguous. Another time he asks Ben to repeat the possible answers. Ben gives stage directions, e.g. you can give more than one answer, and he reads out Mr Friedemann’s ongoing grade which changes after each answer.

Anton and Johannes are watching quietly but look on expectantly every time the teacher answers a question. When it comes to an economic question which includes the term subsistence, Ben gets muddled. He can’t pronounce the word correctly despite repeated attempts. Mr Friedemann, who in the meantime seems to be a bit annoyed by the quiz and especially by the way the question is being asked, wants to read for himself. He is leaning his upper body on the table by this point, and seems somewhat resigned. No no, he won the reading competition, he can read fine, Ben says and tries one last time. Mr Friedemann gives his answer to the question. The whole time Ben keeps the tablet in his hand.

Then Mrs Oras, the other class tutor, enters the room and stands next to me for a short while, also watching the scene. For a moment, I cannot see what is happening. When my field of vision is free again, I hear Ben call out, ‘Now you can still get to 1.9!’ Mr Friedemann formulates his answer. ‘You have a 2.0.’ Mr Friedemann wants to know what kind of quiz it actually is. ‘Schlaukopf. German History Basic Knowledge, Advanced Level.’ Ben answers and adds, ‘so you would have passed the G-course [Grundkurs, i.e. foundation level]’. (Fieldnote_class_35)

The situation exudes playfulness. Ben shows himself to be cheeky and charming, inviting Mr Friedemann into a game situation. The students are not trying to show their teacher up; the other students, Anton and Johannes, look on expectantly; Ben encourages Mr Friedemann at the end that he can still get a 1.9 (equivalent to an A- in other educational systems). And the teacher joins in, somewhat wearily, but he is being a good sport and joins the game in the role (learner, test-taker) that he has been assigned. Ben does not directly take on the inverse role of tester here; using his voice and bodily posture, and not letting go of his tablet or his speaking rights, even when Mr Friedemann tries to take over, Ben becomes the moderator. It is not Ben himself who is testing and evaluating Mr Friedemann, it is the system that is generating the questions, deciding if the answer is right or wrong and calculating the grade. This externalisation of the test situation, and in particular of the grading, to an automated system makes use of automation’s more general promise of objectivity.

The grades, which Ben calls out after each question, symbolise ability: Is the teacher able to get consistently high grades throughout the series of questions? Grading, and the demonstration of ability, unfold in this scene within several specific contexts. Firstly, in this school, quantified grading is only introduced in year nine. This is the first year that these students have received numerical grades on a scale of 1–6 (equivalent to A, B, C, etc.) rather than written feedback. Secondly, this particular project day is explicitly oriented to the students practising tests, to testing themselves and being tested. They are invited on this day to evaluate themselves, using a range of media, and to consider the consequences for their future learning paths as they head towards more high-stakes tests at the end of year ten. Thirdly, the playfulness of the scene hints at the specific relations between students and tutors in this school. Each class has two tutors who accompany the class from Grade 5 to Grade 10. They teach specific subjects (here: history), but they have dedicated time available for social activities and to engage with the students’ well-being, family issues, etc. which in our observations meant that the tutors also shared a good deal of personal details about their lives, families, etc. with their students. Markus Friedemann and Carla Oras are thus embedded in relations with their students that go beyond a perhaps ‘classic’ teacherly position, with a longer history of developing mutual trust.

These specific contexts matter in how something is enacted as automation. Automation becomes visible here as deeply embedded in voice, bodily posture, the holding of devices, struggles over the right to voice and the position of the seats and the desks in long-term student–teacher relationships. The interweaving of these elements together with (*sym-*) a specific website, with a specific set of algorithms is what enacts something that seems to be automatic in a decidedly symmatic way.

The symmation reworks ostensibly hierarchical relations between teachers and students, and between high-stakes educational test systems and students: following Victor Turner’s idea of liminality or liminoid situations as situations of voluntarily chosen phases of transition (Turner 1982; Ball and Savin-Baden 2022), grading Mr Friedemann is an intermittent reversal of roles and responsibilities. The symmated grading reworks the relations between students and teachers and testing precisely because of its seemingly personalised, automated, objective, external and individualised real-time feedback in the form of grades. It enables the students and the teacher to unravel the relations, re-distribute roles and assemble classroom relations in a new way. At the same time, Mr Friedemann’s resistance and attempts to remain the one who knows show that it is a gentle and fleeting shift rather than a revolutionary change.

## Concluding Thoughts: Observing Automation and Identifying Symmation

Though the relation between automation and education has been analysed critically at the level of systems and platform logics, the small and apparently mundane aspects of automation in the everyday lives of students and teachers have been investigated less frequently. As noted above, we set out to observe what people—especially students and teachers—do with *automation* in school settings, and found scenes of what we are calling *symmation*, the ‘making-with’ of automation across bodies, voices, desks, mobile devices, cursors, algorithms, websites, sighs, chairs, apps, playfulness, weariness, languages, gazes and much more. The long-term ethnographic approach of this paper brings these small, unspectacular practices of enacting auto-/symmation—in front of the screen—into view. Yet the ostensibly unspectacular can have far-reaching implications for our understanding of the power of automation to configure education. It rubs the potential and promise of automation against the everyday practices and relations of doing automation (or rather: doing symmation) in educational spaces.

The paper teased out three ways in which auto-/symmation was enacted and in doing so carved out automation’s deep involvement in social relations: *Re-alignment* refers to the often overlooked practices of working *with* Google’s autocomplete to find a search term that will find the relevant information. In this scene, perhaps the most minor in this paper, we see an everyday practice that may resonate with most readers. In a struggle with autocomplete over the ability to define the search, the student repeatedly realigns the search away from the system’s predictions. Together, student and autocomplete thus symcomplete the search. The act of *revising* highlights how automated processes pose a challenge to pedagogical settings, but in turn are embedded into existing relationships of care and respect. Before she understands how the mistakes are automatically generated, the teacher reads the repeated mistakes in her student’s work as peculiar, given her assessment of his abilities. Contrasting the promises and critiques of automation for education, her labour and his learning become neither more machine-like nor more efficient and effortless. The sociomaterial ‘with’ of symmation across student–teacher-algorithmic-linguistic enactments create the student’s presentation in its final, still incorrect, form. The *reworking* of relations in the final scene momentarily upends traditional expectations of teacher-student hierarchies and their respective roles in test-giving and test-taking, in grading and being graded. Cheekiness, charm and playacting work together with (*sym-*) websites, algorithms, desks and chairs to give the teacher real-time grades on his performance of knowing about history.

In sum, the scenes analysed here point out how ability, difference and hierarchies—as essential aspects of sociality in the classroom—are reworked through symmation. Ways of knowing as well as ways of relating to each other shift slightly. However, the scenes are not in the first instance scenes of ‘resistance’ or ‘subversion’ (Perrotta et al. 2020); these are not activist communities that deliberately usurp the logics of automation (as described by Smith and Fressoli 2021). They are also not necessarily best described as the ‘workarounds’ or ‘care’ for technical systems which other close analyses have observed in other schools’ work with data and/or automation (Selwyn 2022a; Zakharova and Jarke 2022). In each of these studies, the ‘thing’ which becomes automation is described (in interviews) or enacted (in practice) as something different. This is perhaps the key point of close analyses of practices: That automation is inevitably different for different students and different teachers with different technologies in the different classrooms of different schools. Identifying specific, situated differences offers a critique of broad statements about the general benefits or challenges of increased automation in schools, often voiced in the edtech industry, in reports from practice or in scholarship. Instead, these close observations hope to shift the terms of debate on automation in education by opening up ‘ways of theorising and practising digital education and automated teaching which are driven neither by technical-rational efficiency models, nor by equally instrumentally focused social models which assume a position of humanistic opposition to or appropriation of digital technology’ (Bayne 2015, referring to Feenberg 2003). Students and teachers are not only using these automated technologies, the technologies are part of the entangled sociomaterial configurations which shape students and teachers.

In the scenes of this paper, we have identified a sociomaterial enactment of auto-/symmation. We could draw here on a vocabulary that describes automation as enrolled or mobilised in these scenes. This vocabulary can still, however, imply an ontology in which automation is stable, ‘out there’, something which exists on its own, and can thus be ‘used’, ‘integrated’, ‘enrolled’ or ‘mobilised’. With the notion of symmation, we have reflected in this paper on what happens if we push this one step further. What becomes visible is how students and teachers muddle along, how they primarily orient to social relations, how they playfully or purposefully reassert—together with the automated systems and platforms—a sociality which exceeds the plans and purposes that corporations, platforms or automated decision-making systems may bring to public education.

These everyday practices create friction in the ‘friction-eliminating logic’ of automated services that aim to help us avoid multifaceted human interactions and instead reduce interaction to ‘seamless’, ‘low-risk’ encounters (critical analysis in Gilliard 2018). And it is precisely friction that enables the ‘grip of encounter’, the grip that lets the wheels turn. Friction, as a metaphor, ‘reminds us that heterogeneous and unequal encounters can lead to new arrangements of culture and power’ (Tsing 2005: 5).

What this friction looks like in schools with more advanced ‘high-level’ automated systems, e.g. systems like AI-driven predictive analytics, is a matter for future ethnographically inspired research. While these systems are not yet very widespread in schools, they are nevertheless a mundane part of everyday life for those engaging with them. In addition, for a fuller picture of how to make sense of auto-/symmation in education, research should return to corporations such as Google or other leading (edtech) providers to focus ethnographically on automation-in-the-making, i.e. focusing on the frictions that arise during the work of rendering processes ‘seamless’.

Ethnographic studies often end with the conclusion that ‘it’s complicated’. Because when ethnographers look closely, it invariably is. The question is then where, in what way, and for whom everyday automation in educational settings is complicated. Google dominates this study, as it does many other contemporary studies of technologies in education. The way multinational corporations configure education needs deep critical analysis. At the same time, the messy practices of exceeding these configurations also require in-depth, close-up, and ‘slow’ analysis (Mountz et al. 2015). This close look led us to reflect on specific practices of realigning, revising and reworking relations, and to suggest symmation as a concept for the situated sociomaterial intricacies of enacting automation. Automation becomes a different thing, enacted differently, with different entanglements in hierarchies, social relations or normativities, depending on the situation. As the vignettes show, automation can be a substitute for work which could be done by students or teachers themselves. Automated processes might fit well into the logic of the task, but might also cause problems when challenging the existing rules and routines. This points to a redistribution rather than a reduction of work, which goes along with a redistribution of responsibility for the working process. Automated processes relieve individual participants with regard to various practices such as searching for information or implementing the right spelling. But they only do this in specifically framed situations, or more precisely: in situations into whose rules and norms they fit. For the classroom, a certain friction remains. It is this friction that brings forth the fragility of automation’s supremacy.

A final point to consider is how to refer to automation. Our ethnographic explorations suggest not thinking of automation as something outside the situation that is unfolding. Enacting automation is rather a ‘sym-mation’ in the sense of ‘with’ than an ‘auto-mation’ in the sense of ‘self’, on one’s own: This, the main argument of the paper, is therefore an attempt to decentralise automation in education. Not to attribute supremacy to the process of automation, but to focus instead on the mutual enactment of auto-/symmation in class, leads to further questions about the ongoing processes of automation, datafication and platformisation in education in particular, and postdigital classrooms in general.

## Footnotes

1 See also http://neveralonegame.com/. Accessed 21 October 2022.

2 The names of all people, institutions and places are pseudonyms.

## References

[CR1] Agar, M. (2006). An Ethnography By Any Other Name... *Forum Qualitative Sozialforschung, 7*(4). 10.17169/fqs-7.4.177.

[CR2] Bastani, A. (2019). *Fully Automated Luxury Communism*. *A Manifesto*. New York and London: Verso.

[CR3] Ball, J., & Savin-Baden, M. (2022). Postdigital Learning for a Changing Higher Education. *Postdigital Science and Education*, *4*(3), 753-771. 10.1007/s42438-022-00307-2. 10.1007/s42438-022-00307-2 PMC9058750 40477619

[CR4] Bayne, S. (2015). Teacherbot: Interventions in automated teaching. *Teaching in Higher Education*, *20*(4), 455–467. 10.1080/13562517.2015.1020783.

[CR5] Breidenstein, G., Hirschauer, S., Kalthoff, H., & Nieswand, B. (Eds.). (2020). *Ethnografie. Die Praxis der Feldforschung*. Konstanz: C. H. Beck.

[CR6] Cameron, P., MacLeod, J., Tummons, O., & Ajjawi, J. (2020). Unpacking practice. The challenges and possibilities afforded by sociomaterial ethnography. In J. Lynch & J. Rowlands, (Eds.), *Practice Methodologies in Education Research* (pp. 187–205). Oxford and New York: Routledge.

[CR7] Cone, L. (2021). The Platform Classroom: Troubling Student Configurations in a Danish Primary School. *Learning, Media and Technology.*10.1080/17439884.2021.2010093.

[CR8] Dixon-Román, E., Nichols, T. P., & Nyame-Mensah, A. (2020). The racializing forces of/in AI educational technologies. *Learning, Media and Technology*, *45*(3), 236-250. 10.1080/17439884.2020.1667825.

[CR9] Feenberg, A. (2003). Modernity Theory and Technology Studies: Reflections on Bridging the Gap. In T. J. Misa, P. Brey, & A. Feenberg (Eds.), *Modernity and Technology* (pp. 73-104). Boston, MA: MIT Press.

[CR10] Gallagher, M., & Breines, M. (2021). Surfacing knowledge mobilities in higher education: Reconfiguring the teacher function through automation*. Learning, Media and Technology*, *46*(1), 78–90. 10.1080/17439884.2021.1823411.

[CR11] Gilliard, C. (2018). Friction-free racism. Real Life, 15 October. https://reallifemag.com/friction-free-racism/. Accessed 5 May 2022.

[CR12] Haraway, D. (2016). *Staying with the Trouble. Making Kin in the Chtulucene.* Durham, NC: Duke University Press.

[CR13] Hartong, S., & Förschler, A. (2019). Opening the black box of data-based school monitoring: Data infrastructures, flows and practices in state education agencies. *Big Data & Society*, *6*(1), 1-12. 10.1177/2053951719853311.

[CR14] Jandrić, P., Ryberg, T., Knox, J., Lacković, N., Hayes, S., Suoranta, J., Smith, M., Steketee, A., Peters, M., McLaren, P., Ford, D. R., Asher, G., McGregor, C., Stewart, G., Williamson, B., & Gibbons, A. (2018). Postdigital Dialogue. *Postdigital Science and Education, 1*(1), 163-189. 10.1007/s42438-018-0011-x.

[CR15] Jarke, J., & Macgilchrist, F. (2021). Dashboard stories: How the narratives told by predictive analytics reconfigure roles, risk and sociality in education. *Big Data & Society, 8*(1), 1-15. 10.1177/2053951721102556.

[CR16] Law, J., Afdal, G., Asdal, K., Lin, W., Moser, I., & Singleton, V. (2013). *Modes of Syncretism: Notes on Non-Coherence*. CRESC Working Paper 119. http://www.cresc.ac.uk/publications/modes-of-syncretism-notes-on-non-coherence. Accessed 21 October 2022.

[CR17] Macgilchrist, F. (2012). E-Schulbücher, iPads und Interpassivität: Reflexionen über neue schulische Bildungsmedien und deren Subjektivationspotential. *bildungsforschung*, *9*(1), 180–204. http://bildungsforschung.org/index.php/bildungsforschung/article/view/151.

[CR18] Macgilchrist, F. (2021). Theories of Postdigital Heterogeneity: Implications for Research on Education and Datafication. Postdigital Science and Education , 3(3), 660-667. 10.1007/s42438-021-00232-w 10.1007/s42438-021-00232-w PMC8122191 40477415

[CR19] Macgilchrist, F., Jornitz, S., Mayer, B., & Troeger, J. (2022). Adaptive Lernsoftware oder adaptierende Lehrkräfte? Das Ringen um Handlungsspielräume. In A. Bock, A. Breiter, S. Hartong, J. Jarke, S. Jornitz, A. Lange, & F. Macgilchrist (Eds.), *Die datafizierte Schule*. Wiesbaden: Springer VS.

[CR20] Manovich, L. (2013). *Software takes command: Extending the language of new media*. London: Bloomsbury.

[CR21] Mountz, A., Bonds, A., Mansfield, B., Loyd, J., Hyndman, J., Walton-Roberts, M., Basu, R., Whitson, R., Hawkins, R., Hamilton, T., & Curran, W. (2015). For Slow Scholarship: A Feminist Politics of Resistance through Collective Action in the Neoliberal University *ACME: An International E-Journal for Critical Geographies*, *14*(4), 1235 - 1259.

[CR22] Perrotta, C., Gulson, K. N., Williamson, B., & Witzenberger, K. (2020). Automation, APIs and the distributed labour of platform pedagogies in Google Classroom. *Critical Studies in Education, 62*(1), 97-113. 10.1080/17508487.2020.1855597.

[CR23] Pink, S., Berg, M., Lupton, D., & Ruckenstein, M. (2022). *Everyday Automation: Experiencing and Anticipating Emerging Technologies*. Abingdon: Routledge. 10.4324/9781003170884.

[CR24] Popenici, S. A. D., & Kerr, S. (2017). Exploring the impact of artificial intelligence on teaching and learning in higher education. *Research and Practice in Technology Enhanced Learning*, *12*(1), 22. 10.1186/s41039-017-0062-8. 30595727 10.1186/s41039-017-0062-8 PMC6294271

[CR25] Rabenstein, K. (2018). Ding-Praktiken. Zur sozio-materiellen Dimension von Unterricht. In M. Proske & K. Rabenstein (Eds.), *Kompendium Qualitative Unterrichtsforschung. Unterricht beobachten – beschreiben – rekonstruieren* (pp. 319–347). Bad Heilbrunn: Klinkhardt.

[CR26] Rabenstein, K., Macgilchrist, F., Wagener-Böck, N., & Bock, A. (2022a). Lernkultur im digitalen Wandel. Methodologische Weichenstellungen einer ethnografischen Fallstudie. In C. Kuttner & S. Münte-Goussar (Eds.), *Medienbildung und Schulkultur. Praxistheoretische Perspektiven auf Schule in einer Kultur der Digitalität* (pp. 179–196). Wiesbaden: Springer.

[CR27] Rabenstein, K., Wagener-Böck, N., Macgilchrist, M. & Bock, A. (2022b). Interferenzen in digitalen Praktiken der Bereitstellung von unterrichtlichen Aufgaben. Ethnographische Beobachtungen in der Pandemie. *Sozialer Sinn, 23*(2), 297–315. 10.1515/sosi-2022-0016.

[CR28] Röhl, T. (2015): Transsituating Education. Educational Artefacts in the Classroom and Beyond. In S. Bollig, M.-S. Honig, S. Neumann, & C. Seele (Eds.), *MultiPluriTrans in Educational Ethnography. Approaching the Multimodality, Plurality and Translocality of Educational Realities* (pp. 121–140). Bielefeld: transcript.

[CR29] Schatzki, T. R. (1996): *Social practices: A Wittgensteinian approach to human activity and the social.* Cambridge: Cambridge University Press.

[CR30] Selwyn, N., & Jandrić, P. (2020). Postdigital Living in the Age of Covid-19: Unsettling What We See as Possible. *Postdigital Science and Education, 2*(3), 989-1005. 10.1007/s42438-020-00166-9. 10.1007/s42438-020-00166-9 PMC7344032 40477067

[CR31] Selwyn, N., Hillman, T., Bergviken Rensfeldt, A., & Perrotta, C. (2021). Digital Technologies and the Automation of Education — Key Questions and Concerns. *Postdigital Science and Education*. 10.1007/s42438-021-00263-3.

[CR32] Selwyn, N. (2022a). ‘Just playing around with Excel and pivot tables’ - the realities of data-driven schooling. *Research Papers in Education, 37*(1), 95-114. 10.1080/02671522.2020.1812107.

[CR33] Selwyn, N. (2022b). Less work for teachers? The ironies of automated decision-making in schools. In S. Pink, M. Berg, D. Luption, & M. Ruckenstein (Eds.), *Everyday Automation: Experiencing and Anticipating Emerging Technologies *(pp. 73–86). Abingdon: Routledge.

[CR34] Sims, C. (2017). *Disruptive Fixation. School Reform and the Pitfalls of Techno-Idealism*. Princeton, NJ: Princeton University Press.

[CR35] Smith, A., & Fressoli, M. (2021). Post-automation. *Futures*, *132*, 102778. 10.1016/j.futures.2021.102778.

[CR36] Sullivan, D. (2018). How Google autocomplete works in Search. https://blog.google/products/search/how-google-autocomplete-works-search/. Accessed 21 October 2022.

[CR37] Tsing, A. L. (2005). *Friction. An Ethnography of Global Connection*. Princeton, NJ: Princeton University Press.

[CR38] Turner, V. (1982). *From Ritual to Theatre: The Human Seriousness of Play*. New York: PAJ Publications.

[CR39] Williamson, B. (2020). Bringing up the bio-datafied child: scientific and ethical controversies over computational biology in education. *Ethics and Education, 15*(4), 444-463. 10.1080/17449642.2020.1822631.

[CR40] Zakharova, I., & Jarke, J. (2022). Educational technologies as matters of care. *Learning, Media and Technology*, *47*(1), 95-108. 10.1080/17439884.2021.2018605.

